# Impact of the COVID-19 Lockdown on a Long-Term Care Facility: The Role of Social Contact

**DOI:** 10.3390/brainsci11080986

**Published:** 2021-07-26

**Authors:** Arturo X. Pereiro, Carlos Dosil-Díaz, Romina Mouriz-Corbelle, Silvia Pereira-Rodríguez, Ana Nieto-Vieites, Sacramento Pinazo-Hernandis, Carolina Pinazo-Clapés, David Facal

**Affiliations:** 1 Department of Developmental Psychology, University of Santiago de Compostela, 15782 Santiago de Compostela, Spain; arturoxose.pereiro@usc.es (A.X.P.); carlos.dosil@usc.es (C.D.-D.); romina.mouriz@usc.es (R.M.-C.); ananieto.vieites@usc.es (A.N.-V.); 2 Gerontological Therapeutic Complex “A Veiga”, Serge Lucense, 27360 Láncara, Spain; terapia_aveiga@serge.es; 3 Department of Social Psychology, Universitat de Valencia, 46010 Valencia, Spain; sacramento.pinazo@uv.es; 4 Department of Developmental Psychology, Universitat de Valencia, 46010 Valencia, Spain; carolina.pinazo@uv.es

**Keywords:** SARS-CoV-2, crisis, nursing homes, older adults, social isolation

## Abstract

(1) Background: Long-term care facilities (LTCFs) have been harmed by the coronavirus, and older adults have remained isolated for a long time with many restrictions. The aim of this study was to measure the decline in cognitive, functional, and affective status in a care facility after the lockdown in the first wave of the COVID-19 pandemic and to compare it with previous measures in order to determine if this decline was accelerated. (2) Methods: Ninety-eight participants were recruited. Data from three retrospective pre-lockdown assessments and an additional post-lockdown assessment were analyzed. Mixed ANOVA analyses were performed according to the Clinical Dementia Rating levels, considering social-contact frequency during the lockdown as a covariate. (3) Results: The cognitive and functional scores were lower and depression scores were higher after the strict lockdown, accelerating a general pattern of decline that was already present in LTCF residents. The frequency of social contact eliminated the measurement differences in the cognitive and functional scores and the group differences in depression scores. (4) Conclusions: The effects of the SARS-CoV-2 lockdown in an LTCF were mediated by the frequency of contact. Clinical implications: Preventive measures must be taken to ensure social contact with relatives and friends and reduce the negative consequences of social isolation in LTCFs.

## 1. Introduction

Approximately ten years after the H1N1 pandemic, the world has faced the second worldwide pandemic of the 21st century. Its rapid spread forced governments to take drastic lockdown measures, which undoubtedly have saved many lives, but drastically impacted the daily lives of the population, forcing citizens to remain isolated unless it was necessary not to do so. The perceived social isolation during the COVID-19 pandemic had significant psychosocial consequences [1]. In Spain, the strict restrictions on movement and the need to keep distance from one another prohibited family visits, accompaniment, sporadic social contacts, walks, physical exercise routines, and all daily activities outside the home [2]. 

Long-term care facilities (LTCFs) have been especially affected by the coronavirus, with a disproportionately high COVID-19-related mortality [2,3], and the effects of quarantine were more notable, as the residents remained isolated for longer and had more restrictive measures due to the higher health risks. The possibilities for talking about fears, worries, and, in general, about everyday issues were severely reduced [3]. According to Boamah, Weldrick, Lee, and Taylor [4], the risk factors of social isolation in LTCFs included individual (e.g., communication difficulties, cognitive impairment), systemic (e.g., location of the facility, type of services), and structural factors (e.g., socioeconomic status, discrimination). The social distancing measures adopted to prevent the spread of COVID-19 in LTCFs have had potential effects on all three levels.

In Spain, LTCF residents’ profiles are characterized by high levels of dependency and moderate to severe levels of cognitive impairment [5]. Calero and Navarro found significant differences in quality of life and levels of dependency according to the cognitive status of the residents [6]. Regarding the impact of COVID-19 in Spanish LTCFs, a study in the province of Albacete showed pooled excess mortality rates for the first month and three first months of the outbreak of 564% and 315%, respectively, and a higher risk of suffering from the disease was linked to older age, frailty, ambulation problems, and disability [7].

The effect of the social isolation added to the restrictions on therapeutic activities, which might have increased the decline of the functional and cognitive status of the residents, in addition to worsening their affective state. Older adults have expressed concern about their families and the effects of the pandemic.

Loneliness, isolation, and a lack of regular social contact may increase bad moods for those who already live with depression and for those who have not experienced it previously. Depression is negatively associated with health problems. The aims of this paper are: (1) to measure the cognitive, functional, and affective declines in a care facility after the lockdown in the first wave of the COVID-19 pandemic and (2) to explore the role of the lack of social contact during the lockdown in these declines. We hypothesized that the declines caused by the lockdown were greater than those in cognition, functioning, and mood that were already present in pre-COVID-19 circumstances, and that they were greater in residents with lower cognitive status. We also hypothesized that the frequency of social contact during lockdown was relevant for these differences, so introducing a covariate would make the differences that were present without it disappear.

## 2. Materials and Methods

### 2.1. Participants

Ninety-eight participants aged 60 years and older were recruited from residents of the “A Veiga” Gerontological Therapeutic Complex, which is located in a rural area of province of Lugo (Galicia, Spain). This care center offers 176 places for old and disabled persons, of which 60% are subsidized by the autonomous government (Xunta de Galicia). Medical and nurse attention, psychological interventions, occupational therapy, and several residential services are provided. The following inclusion criteria were applied: (1) they had stayed in the care facility for the entire period of confinement (three months); (2) they were 60 years old or older; (3) three measurements prior to the lockdown period were available. Of the 170 residents in the center in March 2020, 8 were not included in the study because they or their legal representatives refused to participate, 8 were not included because they did not stay in the care facility for the entire period of confinement, 5 were not included because they were under 60 years old, and the rest who were excluded were left out because not all of the required pre-lockdown assessments were available.

The participants and/or their legal representatives were informed about the study, its procedure, its voluntary nature, and the measures taken to ensure their privacy according to Spanish and European laws. Informed consent forms were signed by the participants and/or their legal representatives. They were informed that, although they were giving consent to participate in this study, they retained their right to retract and abandon the study at any time that they wished.

### 2.2. Instruments

Cognitive status was measured with the Spanish version of the MMSE [8]. The MMSE is a widely used cognitive screening instrument whose consistency in its Spanish version varies between 0.82 and 0.84, and its inter-judge reliability varies between 0.83 and 0.99 in elderly people and patients with neurological diseases. Depressive symptomatology was measured with the Spanish version of the 15-item version of the Geriatric Depression Scale (GDS) [9]. The GDS is a short instrument designed to assess depressive dysfunctions in adults who are older than 65 years. The intra-observer reliability of the 15-item Spanish version is 0.95, its inter-observer reliability is 0.65, and its internal consistency is 0.99. Because it is a self-reported test, the GDS was not considered for participants who had a CDR value of 3. It is a self-reported test in which the elderly adults provided information about their affective state by using a Likert-type response scale. In participants with severe cognitive impairment (CDR = 3), the responses were not reliable and, subsequently, were not analyzed when we studied the depressive symptomatology. For other analyses that did not use self-reported information, the participants with CDR = 3 were included. The functional status was measured with the Spanish version of the Barthel Index (BI) [10]. The BI is the most commonly employed questionnaire for the evaluation of functionality in the Spanish context, although its psychometric properties are less well known. Gonzalez et al. obtained Cronbach’s alpha coefficients that were greater than 0.87, and a confirmatory factor analysis supported its unidimensionality [11]. In each of the three pre-lockdown evaluations and in the post-lockdown evaluation, the same evaluation instruments were used.

A Spanish version of the Clinical Dementia Rating (CDR) [12] was used to establish participants’ cognitive status at baseline. The CDR is a semi-structured interview designed for use with elderly adults and an informed proxy, and it rates impairment in six categories (memory, orientation, judgment, and problem solving, community affairs, home and hobbies, and personal care). As a clinical assessment of cognitive impairment, the CDR is independent from psychometric tests that measure cognitive and functional performance. In the Spanish context, the levels of cognitive impairment and comorbidity are high in LTCFs [5,6], and clinical staging instruments for dementia are commonly used to classify the residents and organize their care.

The participants’ sociodemographic information, health, and lockdown circumstances (place, social contact, leisure, therapies) were evaluated with an ad hoc questionnaire that was rated by the psycho-gerontologist and the occupational therapist. Social-contact frequency during the lockdown was studied with the question “The person was able to communicate with their family and friends by phone or other telematic means” with the following scale: 1 = without contact; 2 = biweekly/monthly; 3 = weekly; 4 = daily. All of the available modalities of social contact (phone calls, video calls, chat) with family and friends outside the LTCF were considered. It was decided that the information on lockdown circumstances would be collected by the center’s professionals in consideration of the diversity in the cognitive status of the residents and the fact that there was a detailed record of these circumstances during lockdown. For example, in relation to social contact, the confinement conditions made spontaneous contact impossible for most of the residents, especially those with cognitive impairments who used to receive visitors before the pandemic, and social contact began to be promoted and registered by the professionals through the center’s phones and tablets.

### 2.3. Procedure

Three retrospective pre-lockdown measures of cognitive status, depression symptomatology, and functional status were recovered from the records of the center, which were managed through ResiPlus, a software that was specifically designed for long-term care institutions in order to enable comprehensive data management and that is widely implemented in Spanish gerontological centers (https://www.resiplus.uk/software/, accesed on 30 August 2020). All pre-lockdown measurements were carried out in compliance with the institutional evaluation protocol established for the biannual follow-up of the residents. The first pre-lockdown assessment was carried out between December 2018 and June 2019; the second pre-lockdown assessment was carried out between June 2019 and January 2020; the third pre-lockdown assessment was carried out between January and March 2020. On 14 March 2020, the Government of Spain declared a “state of alarm” throughout the territory with measures that placed severe restrictions on the movement of persons and economic activities. In the autonomous community of Galicia, this “state of alarm” lasted until 15 June.

In order to study the effects of the strict lockdown on the residents, an additional prospective post-lockdown measurement was carried out between two weeks and three months after the relaxation of the restrictions of the Spanish and Galician governments in nursing homes (during the period from July to September 2020). Sociodemographic information, health, and lockdown circumstances were also collected at that time. 

The three pre-lockdown evaluations and the post-lockdown evaluation were all conducted by the center’s interdisciplinary team, including professionals in psychology, occupational therapy, medicine, nursing, and social work. 

### 2.4. Data Analysis

Mixed ANOVA analyses (repeated-measure general linear model) were performed with SPSS v20 to test group differences according to the CRD levels (0 = None, 0.5 = Questionable, 1 = Mild, 2 = Moderate, 3 = Severe) between the three pre-lockdown and the post-lockdown measurements of cognitive and functional performance while considering or not considering the social-contact frequency during the lockdown as a covariate. To introduce this covariate, we subtracted the variance associated with contact frequency in the intra- and inter-comparisons, and we could indirectly infer the role of this covariate in the inter- and intra-individual differences. In this way, the pre-/post-lockdown differences in each of the levels of the inter-subject factor (CDR levels) could be analyzed alone and once the influence of social contact was controlled. Multiple-comparison post hoc Bonferroni correction was used to evaluate significant pairwise comparisons. The alpha value was established at 0.05 for all analyses. The partial eta squared value (*ŋ_p_^2^*) was reported as an estimate of the effect size in the mixed ANCOVAs.

## 3. Results

The sociodemographic information and lockdown circumstances are described in Table 1.

The descriptive values (means and standard deviations) for MMSE, GDS, BI, and frequency of social contact are shown in Table 2. Decreasing trends were observed across measurements of the cognitive and functional scores, and increasing trends were found for depressive symptomatology.

The mixed ANOVA analyses for testing inter-subject differences (CDR factor) between measurements (measurement factor) for cognitive performance (MMSE) showed that the main effects were from the CDR (*F*(4,88) = 52.42; *p* < 0.001; *ŋ_p_^2^* = 0.704; *Observed-Power* = 1.0) and measurement factors (*F*(3,86) = 7.21; *p* < 0.001; *ŋ_p_^2^* = 0.201; *Observed-Power* = 0.979). A significant interaction between CDR and MMSE was not found. Bonferroni pairwise comparisons showed that the MMSE scores were significantly lower in the third pre-lockdown measurement and in the post-lockdown measurement, and the scores were significantly lower in the mild, moderate, and severe groups than in the normal and questionable CDR levels.

The mixed ANOVA analyses for testing inter-subject differences between the measurements of depressive symptomatology (GDS) showed that the main effects were from the GDS (*F*(3,65) = 3.09; *p* = 0.033; *ŋ_p_*^2^ = 0.125; *Observed-Power* = 0.697). The main effects for the CDR factor and the interaction between the CDR and GDS were not found to be significant. The Bonferroni comparisons showed that the GDS scores were significantly higher in the post-lockdown measurement than in the first pre-lockdown measurement. 

The mixed ANOVA analyses for testing inter-subject differences between the measurements of functionality (BI) showed that the main effects were from the CDR (*F*(4,91) = 35.94; *p* < 0.001; *ŋ_p_*^2^ = 0.612; *Observed-Power* = 1.0) and BI (*F*(3,89) = 4.15; *p* = 0.008; *ŋ_p_^2^* = 0.123; *Observed-Power* = 0.838). The interaction between the CDR and BI was not found to be significant. The Bonferroni comparisons showed that the BI was significantly higher in the third pre-lockdown measurement and the post-lockdown measurement than in the two first pre-lockdown measurements and that the scores were significantly higher in the mild, moderate, and severe groups than in the normal and questionable CDR levels. 

**Figure 1:**
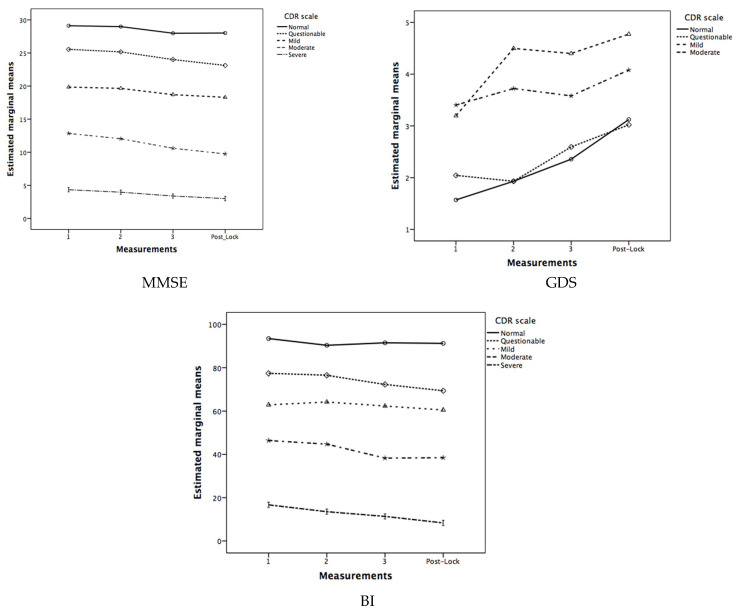
Estimated marginal means for the three pre-lockdown and the post-lockdown Mini-Mental State Examination (MMSE), Geriatric Depression Scale (GDS), and Barthel Index (BI) according to the Clinical Dementia Rating (CDR) levels, and with the social-contact frequency as a covariate.

Regarding the effect of the frequency of social contact during lockdown as a covariate (see Figure 1), the mixed ANCOVA analysis for the MMSE showed that the inter-subject differences according to the CDR were maintained (*F*(4,87) = 44.05; *p* < 0.001; *ŋ_p_*^2^ = 0.669; *Observed-Power* = 1.0), but the intra-subject differences in the measurement factor were not. For the GDS, in the mixed ANCOVA, the significance of intra-subject differences was not achieved. Finally, for the BI, inter-subject differences were also maintained (*F*(4,90) = 31.81; *p* < 0.001; *ŋ_p_*^2^ = 0.586; *Observed-Power* = 1.0), but intra-subject differences in the BI scores were not.

## 4. Discussion

The cognitive and functional scores were significantly lower in the mild, moderate, and severe groups than in the normal and questionable CDR levels, and the scores significantly decreased in the third pre-lockdown measurement and in the post-lockdown measurement. When the frequency of social contact during the lockdown was included in the analysis as a covariate, the significance of the measurement differences disappeared. In particular, depressive symptomatology was the only variable that significantly increased in the post-lockdown measurement, but this measurement difference also disappeared when the frequency of social contact was included as a covariate.

Our results show that the impact of the strict lockdown on the cognitive and functional status of the older adults living in the LTCF did not significantly alter the general pattern of age-related decline observed in the pre-lockdown trends in the context of high levels of dependency and cognitive impairment in Spanish LTCFs [5,6,7]. Interestingly, the frequency of social contact during the lockdown eliminated the differences between assessments; the only post-lockdown effect observed, that of depressive symptomatology, showing a strong association of social contact with the mental health and functional measurements. This frequency of social contact during lockdown could be illustrative of social resources and their continuous influence on mental health and functionality. Social support shown to be a strong protector against the adverse cognitive effects of anxiety and chronic stress [13].

Preventing the spread of COVID-19 to care facilities has been a priority. Our results highlighted the relevance of the implementation of strategies for reducing social isolation and its negative consequences for residents in LTCFs. For example, assistance should be provided when making telephone and/or video calls with relatives and friends [3]. The social isolation caused by COVID-19 has worsened the existing vulnerabilities of these groups of people, such as residents in LTCFs, especially those with dementia, who make up the majority of nursing home residents; they are already at a disadvantage in terms of health and social contact, thus highlighting the need for mitigatory and health-promotion measures [1]. Considering the lockdown in the LTCFs as a natural experiment and taking into account that the residents were confined to their bedrooms or in restricted rooms near their bedrooms with no contact with other residents and restricted contact with their professional caregivers, the social contact that was programmed by the staff by using information and communication technologies (ICTs) was the most relevant social resource for the residents. Accordingly, the variability came from the availability of their own loved ones outside the LTCF and their cognitive, affective, and social resources for taking advantage of calls, video calls, and/or chats. The effects of this type of social contact should be evaluated in more controlled intervention studies.

The present study used variables that were already being collected by the center before the COVID-19 pandemic as dependent variables. Other psychological variables may also have played a relevant role in coping with the COVID-19 pandemic in LTCFs. Stress and how it affects how one copes with situations of isolation, perceived risk of infection, illness, and death has been assessed in Spanish health personnel [14] and nursing home workers [15], but not in elderly adults living in LTCFs. The COVID-19 pandemic has increased stress in the general population, and it would be expected that this increase in stress has been especially pronounced in vulnerable persons, such as the elderly residents of LTCFs. In Spanish older adults who dwell in communities, research has highlighted the role of psychological resources in the ability to adapt to the lockdown due to the first wave of the pandemic, highlighting the role of resilience and resilient coping styles [16,17]. However, more research is needed in order to investigate the role of psychological resources in the coping mechanisms of old adults living in LTCFs during the pandemic.

The major strengths of this paper are (a) the relevance of its objectives in the context of the global COVID-19 pandemic, which has differentially affected older adults and, especially, institutionalized elderly people, and (b) the longitudinal nature of the study with its focus on impact by including pre-pandemic and post-pandemic results while considering the lockdown in LTCFs as a natural experiment. Despite the fact that the CDR is a clinical measure that is independent from direct cognitive, functional, and affective measures and that it was used in our context to organize the groups according to their care and therapeutic needs, the major limitation of this study was that there was a certain risk of circularity when looking for differences between the groups created by using the CDR. The time interval between the relaxation of the restrictions of the Spanish and Galician governments in nursing homes and the post-lockdown measurements (between two weeks and three months) can be considered wide, and the effects of the confinement could be attenuated by time. To understand these temporal differences, it is important to consider the complexity of health and care during the situation during the first wave of the COVID-19 pandemic and the changes that were necessary in the LTCFs. In this sense, as mentioned above, the lockdown in the LTCFs can be considered as a natural experiment. Other limitations would include the relatively small size of the sample and the fact that it was recruited from a single long-term care center. Future studies should include residents of different centers and from different countries and regions. A multicenter study would allow the exploration of the roles of different variables, such as duration in days, changes in groups due to the reorganization of the center, exposure to the media with news about the pandemic, health incidents, or increased falls. These variables were collected in this study but were not analyzed due to the uniformity of the responses according to the center’s response to the pandemic. According to the complexity of these data, a more complex statistical analysis would also be required. Regarding the measurement of social contact, information about social contact was not routinely collected before the COVID-19 lockdown, but we included in it in the prospective post-lockdown measurement. Furthermore, according to the restricted nature of the social contact within the LTCF during the lockdown and due to the social distancing measures decreed to prevent the spread of COVID-19 in the centers, we only measured social contact with people outside through ICTs. Future studies on the relevance of interventions based on social contact using calls, video calls and/or chats should also consider the frequency of contact with other residents and with the staff of the centers, as well as the social resources of the residents, which could be measured with standardized tests.

## Figures and Tables

**Table 1:** Mean age, educational level, professional category, and lockdown circumstances.

Age	*M* = 83.41 (*SD* = 9.61); Range: 60–102
Sex	62% women
Education	Basic: 45%
	Primary: 39%
	Secondary or higher: 16%
Professional Category	No job/occupation: 10%
	Housewife: 46%
	Unskilled worker: 21%
	Skilled worker/entrepreneur: 23%
Therapeutic Routines	Interrupted: 9%
	Lower frequency: 86%
	Same frequency: 5%
Leisure Resources	Not accessible: 18%
	Partially accessible: 82%
Mobility During the Lockdown	Room: 15%
	Room and next-door instances: 80%
	Floor: 4%
	Whole building: 1%

**Table 2:** Descriptive values (means, standard deviations between brackets) for cognitive status (MMSE scores), depression symptomatology (GDS scores), functional status (BI scores), and social-contact frequency during the lockdown according to participants’ CDR status at baseline.

CDR	Degree 0	Degree 0.5	Degree 1	Degree 2	Degree 3
	(*n* = 10)	(*n* = 20)	(*n* = 19)	(*n* = 23)	(*n* = 26)
MMSE_Pre1	30.00 (4.75)	26.44 (5.21)	19.95 (0.48)	12.48 (6.59)	3.92 (5.45)
MMSE_Pre2	29.50 (4.14)	26.00 (5.62)	19.74 (8.63)	11.70 (5.91)	3.58 (5.53)
MMSE_Pre3	29.40 (4.76)	25.40 (5.30)	18.79 (8.17)	10.26 (5.34)	3.00 (4.70)
MMSE_Post	29.40 (4.76)	24.40 (6.96)	18.37 (8.67)	9.48 (5.24)	2.69 (4.57)
GDS_Pre1	1.78 (1.98)	2.45 (2.74)	3.11 (3.82)	3.04 (4.91)	0.22 (1.04)
GDS_Pre2	2.20 (2.74)	2.50 (2.78)	4.37 (5.04)	3.22 (5.00)	0.00 (0.0)
GDS_Pre3	2.40 (3.47)	3.20 (3.59)	4.26 (4.75)	3.04 (5.02)	0.80 (0.39)
GDS_Post	3.10 (3.69)	3.65 (3.29)	4.63 (4.62)	3.52 (5.01)	0.23 (0.86)
BI_Pre1	94.00 (14.10)	78.25 (21.16)	62.89 (23.88)	45.91 (27.42)	16.15 (23.16)
BI_Pre2	90.00 (23.09)	76.00 (24.09)	64.21 (25.23)	45.00 (27.74)	13.85 (17.28)
BI_Pre3	90.50 (21.53)	70.75 (21.16)	62.37 (24.85)	38.91 (27.95)	12.31 (17.27)
BI_Post	90.50 (16.40)	68.25 (24.07)	60.53 (27.83)	38.91 (27.30)	9.04 (14.07)
Social contact during the lockdown	Without contact: 10.00%	Without contact: 10.00%	Without contact: 26.31%	Without contact: 39.13%	Without contact: 50%
	Biweekly/monthly: 30.00%	Biweekly/monthly: 5.00%	Biweekly/monthly: 15.78%	Biweekly/monthly: 26.08%	Biweekly/monthly: 3.84%
	Weekly: 30.00%	Weekly: 55.00%	Weekly: 52.63%	Weekly: 30.43%	Weekly: 46.15%
	Daily: 30.00%	Daily: 30.00%	Daily: 5.26%	Daily: 4.34%	Daily: 0%

Note: CDR = Clinical Dementia Rating; MMSE = Mini-Mental State Examination; GDS = Geriatric Depression Scale; BI = Barthel Index.

## Data Availability

The data presented in this study are available on request from the corresponding author.
